# *STS* and *PUDP* Deletion Identified by Targeted Panel Sequencing with CNV Analysis in X-Linked Ichthyosis: A Case Report and Literature Review

**DOI:** 10.3390/genes14101925

**Published:** 2023-10-10

**Authors:** Joonhong Park, Yong Gon Cho, Jin Kyu Kim, Hyun Ho Kim

**Affiliations:** 1 Department of Laboratory Medicine, Jeonbuk National University Medical School and Hospital, Jeonju 54907, Republic of Korea; miziro@jbnu.ac.kr (J.P.); choyg@jbnu.ac.kr (Y.G.C.); 2 Department of Pediatrics, Jeonbuk National University Medical School and Hospital, Jeonju 54907, Republic of Korea; kyunim@jbnu.ac.kr; 3 Research Institute of Clinical Medicine, Jeonbuk National University-Biomedical Research Institute, Jeonbuk National University Hospital, Jeonju 54907, Republic of Korea

**Keywords:** *STS* gene, *PUDP* gene, hemizygous deletion, copy-number variation, targeted panel sequencing, MLPA, X-linked ichthyosis

## Abstract

X-linked recessive ichthyosis (XLI) is clinically characterized by dark brown, widespread dryness with polygonal scales. We describe the identification of *STS* and *PUDP* deletions using targeted panel sequencing combined with copy-number variation (CNV) analysis in XLI. A 9-month-old infant was admitted for genetic counseling. Since the second day after birth, the infant’s skin tended to be dry and polygonal scales had accumulated over the abdomen and upper extremities. The infant’s maternal uncle and brother (who had also exhibited similar skin symptoms from birth) presented with polygonal scales on their trunks. CNV analysis revealed a hemizygous deletion spanning 719.3 Kb on chromosome Xp22 (chrX:7,108,996–7,828,312), which included a segment of the *STS* gene and exhibited a Z ratio of −2 in the proband. Multiplex ligation-dependent probe amplification (MLPA) confirmed this interstitial Xp22.31 deletion. Our report underscores the importance of implementing CNV screening techniques, including sequencing data analysis and gene dosage assays such as MLPA, to detect substantial deletions that encompass the *STS* gene region of Xq22 in individuals suspected of having XLI.

## 1. Introduction

Ichthyosis is a condition characterized by the excessive growth of the outermost layer of the skin, the stratum corneum, leading to dry skin with scaling. Congenital ichthyosis, which presents during the neonatal period, often poses significant health risks, as the skin’s protective function is compromised [1]. Syndromic ichthyosis is classified based on its genetic origin, including ichthyosis vulgaris, which is inherited in an autosomal dominant manner, and X-linked recessive ichthyosis (XLI). XLI occurs in approximately 1 in 1500 to 6000 males and typically manifests within the first three months of life. Clinically, XLI is characterized by dark brown, widespread dryness with polygonal scales. These cutaneous symptoms appear shortly after birth and tend not to improve with age. The affected areas primarily include the neck, trunk, and extensor side of the limbs. Unlike other forms of ichthyosis, XLI also affects the flexion side of limbs [2]. Histopathologically, XLI typically exhibits mild acanthosis and dense hyperkeratosis with a normal granular layer [3].

Several genetic studies of Korean patients with XLI attributed to *STS* mutations have been documented [4,5,6,7]. In this report, we describe the identification of *STS* and *PUDP* deletions using targeted panel sequencing combined with copy-number variation (CNV) analysis in XLI. To our knowledge, this was the first report of Korean XLI screening through targeted panel sequencing with CNV analysis.

## 2. Materials and Methods

### 2.1. Patient

**Figure 1:**
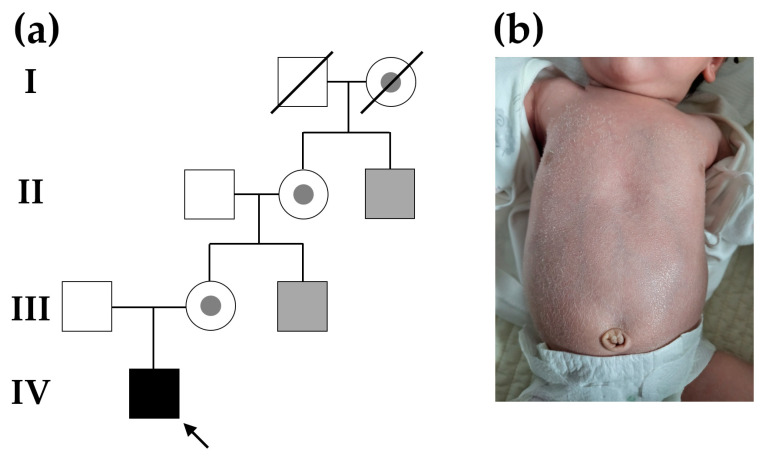
Pedigree analysis and skin image of the proband (indicated by black arrow). (**a**) Family pedigree shows hereditary ichthyosis inherited in an X-linked recessive manner. Carrier status was not genetically confirmed. The asymptomatic mother of the proband was an obligate heterozygote. The gray symbol indicates that the family member was clinically suspected to have ichthyosis but that was not confirmed genetically. (**b**) A 9-month-old infant with ichthyosis due to an *STS* mutation showed abnormal shedding, a tendency toward dry skin, and the accumulation of polygonal scales on the abdomen.

A 9-month-old infant (IV-1 in Figure 1a) was vaginally born at a gestational age of 39 weeks and 5 days. The birth weight was 3300 g, and the 1- and 5-min Apgar score was 10/10, which was good. The mother had no diagnosed diseases before pregnancy or pregnancy-related diseases during pregnancy, and this was her first delivery. The patient visited the Department of Pediatrics at Jeonbuk National University Hospital (Jeonju, Republic of Korea) with skin lesions accompanied by scales all over his body since the second day of birth. The skin tended to be dry, and polygonal scales had accumulated over the abdomen and upper extremities (Figure 1b). Blood count, electrolytes, liver function tests, blood urea nitrogen, creatinine, and C-reactive protein levels were all normal. In the pedigree of three generations, the patient’s maternal uncle had the same skin symptoms as those of the infant, and the other ancestral family members had no skin symptoms. The patient’s maternal uncle and brother, who also exhibited similar skin symptoms from birth, presented with polygonal scales on their trunks. Their symptoms were alleviated within the first year of life by maintaining intensive skin moisturization. They experienced intermittent skin manifestations throughout adulthood, but applying topical moisturizers consistently improved symptoms. Hereditary skin disease was suspected, considering the family history and the disease that occurred early after birth, and related genetic tests were performed. X-linked inheritance was suspected in genetic counseling, because only male family members presented with similar clinical symptoms associated with ichthyosis. G-banding karyotyping showed a normal male karyotype of 46, XY. Then, a targeted panel sequencing was performed that included genes associated with genetic disorders of the skin.

### 2.2. Targeted Panel Sequencing

Genomic DNA extracted from the proband was subjected to analysis using a customized hereditary dermatology panel (Green Cross Genome, Yongin, Republic of Korea). This panel was designed to target the exons and adjacent regions of 95 genes associated with epidermolysis bullosa and ichthyosis (see Table 1 for details). Sequencing was conducted as paired-end (PE) sequencing with a high-output flow cell, involving 300 cycles PE (150 bp × 2) on a NextSeq500 instrument (Illumina, San Diego, CA, USA) at the Green Cross Genome. Data were analyzed following the Genome Analysis Tool Kit best practice pipeline workflow (https://gatk.broadinstitute.org/hc/en-us (accessed on 18 January 2023)), which encompassed processes such as base-calling, base alignment, variant calling, annotation, and quality control reporting.

Medical laboratory geneticists independently reviewed the interpretation of sequence variants, adhering to the standards and guidelines established by the Joint Consensus Recommendation of the American College of Medical Genetics and Genomics and the Association for Molecular Pathology to ensure accuracy [8]. Raw sequencing data generated from targeted panel sequencing underwent CNV analysis using VisCap [9]. In brief, this involved calculating the specimen-specific fractional coverage for each site, which was then divided by the median value for that region across the entire batch. A boxplot interquartile range multiplier of 3 was applied as a cutoff for detecting gains and losses. Specifically, a gain was defined by a minimum log2 ratio of 0.40, while a loss was defined by a maximum log2 ratio of −0.55, based on the distribution of log2 ratios for each specimen.

### 2.3. Multiplex Ligation-Dependent Probe Amplification

We employed SALSA^®^ MLPA^®^ Probemix P160-C1 STS reagent (MRC-Holland, Amsterdam, The Netherlands) to assess large exon deletions in the *STS* gene identified through targeted panel sequencing with CNV analysis. The procedure followed the manufacturer’s protocol for multiplex ligation-dependent probe amplification (MLPA). Subsequently, capillary electrophoresis and fragment analysis were performed consecutively using a 3500XL Dx Genetic Analyzer (Applied Biosystems, Carlsbad, CA, USA). Coffalyser.Net software version 220401.000 (MRC-Holland) was utilized for the comparative analysis of CNV data in accordance with the manufacturer’s instructions. Three sex-matched normal DNA samples were employed to normalize the resulting peak intensities. Manufacturer control probes served as references for the analysis. Generally, probe-to-peak ratios ranging from 0.7 to 1.3 were considered indicative of a normal copy number (wild-type). Conversely, a loss of one copy number was defined by probe-to-peak ratios between 0.4 and 0.7. A gain of one copy number was inferred from probe results for probe-to-peak ratios between 1.3 and 1.6.

## 3. Results

**Figure 2:**
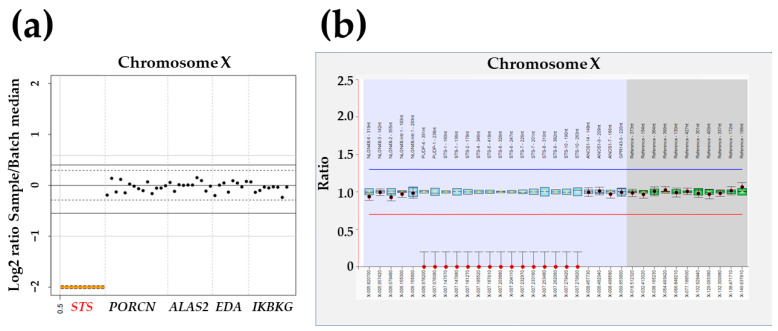
Hemizygous *STS* deletion identified by targeted panel sequencing with copy-number variation (CNV) analysis and multiplex ligation-dependent probe amplification (MLPA) in the proband with X-linked ichthyosis. (**a**) CNV analysis with VisCap illustrated a hemizygous 719.3 Kb deletion in chromosome Xp22 (chrX:7,108,996–7,828,312), including a region of the *STS* gene with a Z ratio of −2 in the proband. (**b**) MLPA confirmed the same hemizygous deletion between exons 1–4 of the *PUDP* gene and the whole region of the *STS* gene screened by CNV analysis.

No (likely) pathogenic variants, such as single nucleotide variants (SNVs) or small indels, were identified through targeted panel sequencing. However, CNV analysis revealed a hemizygous deletion spanning 719.3 Kb on chromosome Xp22 (chrX:7,108,996–7,828,312), which included a segment of the *STS* gene and exhibited a Z ratio of −2 in the proband (Figure 2a). MLPA confirmed this hemizygous deletion, encompassing exons 1 to 4 of the *PUDP* gene and the entire region of the *STS* gene, as identified by CNV analysis in targeted panel sequencing (Figure 2b). Consequently, our proband received a diagnosis of XLI resulting from the deletion of both the *STS* and *PUDP* genes. Unfortunately, other family members declined to participate in the segregation analysis. The patient’s skin symptoms showed partial improvement following regular and intensive moisturization. No deterioration in symptoms was noted at the latest follow-up at 9 months of age.

## 4. Discussion

Genomic instability is a notable characteristic of the human Xp22.31 region. Deletions occurring in this region have been linked to a range of conditions, including XLI, mental retardation, and attention-deficit/hyperactivity disorder (ADHD). This instability is partly attributed to the presence of low-copy repeats (LCRs), which are DNA sequences that are duplicated in the genome. LCRs mediate recurrent deletions at this locus, leading to associated disorders. Additionally, the Xp22.31 region is enriched with a putative homologous recombinant hotspot motif. This motif likely plays a role in facilitating recombination events that lead to these recurrent deletions [10].

Homologous recombination is a genetic mechanism in which similar or identical DNA sequences recombine with one another, potentially leading to DNA rearrangements, such as deletions. The presence of this recombinant hotspot motif in the region may contribute to the genetic instability observed in Xp22.31 and the associated disorders. [10]. Most XLI cases (up to 90%) stem from a deletion that completely encompasses the *STS* gene [3]. Typically, the deletion in Xp22.31 spans approximately 1.6 Mb and includes the *STS*, *HDHD1*/*PUDP*, *PNPLA4*, and *VCX* genes’ encoding proteins. This deletion is classified as pathogenic, based on the recommendations of the American College of Medical Genetics and Genomics [11], and results in ichthyosis, a condition primarily affecting males. 

Our patient visited the hospital with symptoms of ichthyosis vulgaris, and initial genetic testing was performed through targeted panel sequencing according to the symptoms. No (likely) pathogenic SNV or small indel variants were detected. However, CNV analysis using massively parallel sequencing (MPS) data generated from targeted panel sequencing identified a hemizygous 719.3 Kb deletion in chromosome Xp22. Subsequent MLPA confirmed this large deletion, enabling the early diagnosis of a congenital disease caused by *STS* mutations related to recessive ichthyosis in the X chain during the neonatal period. 

Ichthyosis identified early in life shows varying severity depending on the causative disease [1]. If the symptoms are not severe, they can be confused with temporary skin changes in the neonatal period. Therefore, targeted panel sequencing tests can be applied in the neonatal intensive care unit soon after birth if screening tests to exclude syndromic ichthyosis are warranted. Since the panel tests the proven genes according to the symptoms of the patient, it can provide the genetic confirmation of a neonatologist’s clinical diagnosis [12]. Based on symptoms and family history, additional analysis using sequencing depth confirmed a hemizygous deletion in the region, including *STS*, leading to the final diagnosis.

Clinical manifestations are closely linked to the content of neighboring genes. Extensive deletions that encompass a larger number of nearby genes are correlated with severe conditions. These conditions manifest as ocular albinism, epilepsy, abnormal electroencephalographic readings, intellectual disabilities, reduced sense of smell (hyposmia), ADHD, autism, and language development disorders [13,14]. Some male patients may exhibit only minor skin lesions, such as widespread dry and scaly skin and scaling [15,16]. In addition to ichthyosis, benign corneal opacities affect approximately 10–50% of males with XLI [17,18], and roughly 20% of males with XLI experience cryptorchidism [19]. Male carriers of typical XLI-associated deletions approximately 1.6 Mb in size were reported to exhibit developmental delays [20], intellectual disabilities [21], autism [22], epilepsy [21,23], and kidney abnormalities [24]. Despite the established association, the skin symptoms can be considered relatively mild compared to other inherited dermatologic conditions or phenotypes linked to other pathogenic CNVs that might be identified prenatally. Thus, the primary focus is, typically, on neurodevelopmental outcomes and structural anomalies.

Recent studies reported differences from other cases involving Xp22.31 CNVs and attempts to establish correlations between disease severity, the size of the chromosomal deletion, and gene content [25,26]. In clinical and molecular findings in 35 Italian patients [25], the frequency of cryptorchidism (12.9%) corresponded to previous XLI studies. However, the absence of corneal opacities in these individuals can be attributed to their youth. Out of 33 patients, nine (27%) displayed symptoms of ADHD, which aligns with recent literature reporting the heightened occurrence of these behavioral traits in patients with XLI. ADHD has been documented in XLI patients, stemming from both *STS* deletions and point mutations. These findings suggest that *STS* deficiency directly contributed to the development of inattentive and hyperactivity symptoms. In genotype-phenotype correlations in prenatal and postnatal follow-ups of 87 fetuses [26], while skin disorders in male fetuses associated with Xp22.31 deletions could be improved with appropriate treatment, the presence of this deletion in males may also be considered a potential susceptibility factor for neurodevelopmental disorders. 

A literature review of Korean patients with XLI found that six cases were genetically confirmed by chromosomal microarray (CMA) or MLPA. Depending on the size of the Xp22 deletion, only skin symptoms or other combinations of symptoms were seen. The larger the size of the Xp22 deletion, the more diverse and serious symptoms tended to appear in these cases (Table 2). 

**Figure 3:**
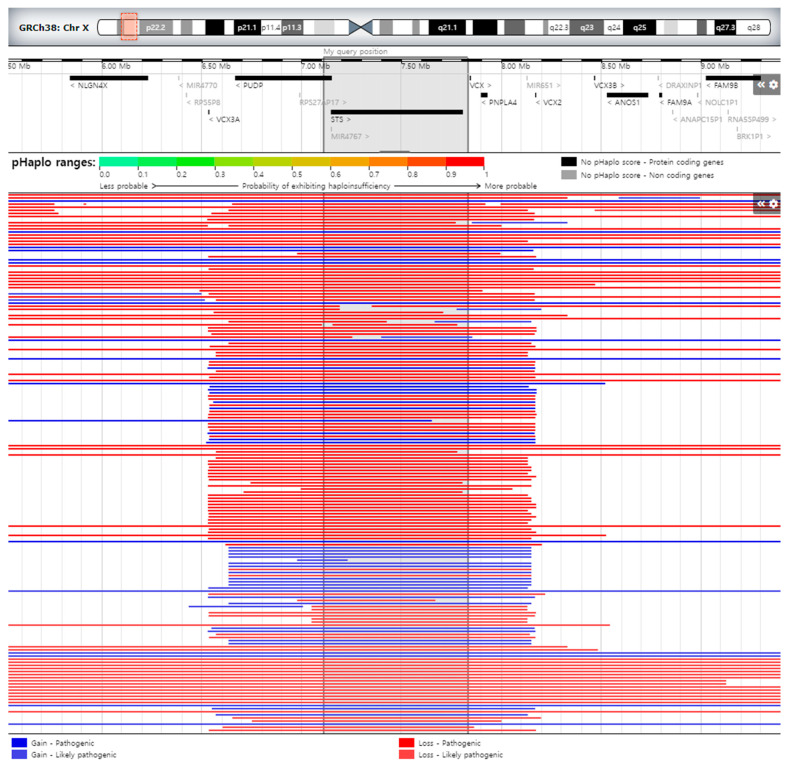
Schematic representation of our patient’s deletion compared to previously reported cases spanning the interstitial 4.4 Mb of chromosome Xp displayed by DECIPHER’s genome browser (https://www.deciphergenomics.org/browser#q/X:5000000-9400000/location/X:4875000-9525000; accessed on 11 September 2023). Red and blue bars indicate pathogenic/like pathogenic losses and gains, respectively, of any size (Mb) by Decipher.

We depicted our patient’s deletion through a schematic representation, which was compared to previously documented cases covering the interstitial 4.4 Mb of chromosome Xp, as visualized using DECIPHER’s genome browser (https://www.deciphergenomics.org/browser#q/X:5000000-9400000/location/X:4875000-9525000; accessed on 11 September 2023) (Figure 3). The skin-barrier function remains intact, and there is a high occurrence of kallikrein 7 gene polymorphism among Korean patients with XLI. Specifically, the histopathological examination of ichthyosis epidermis did not reveal acanthosis, and there were no significant differences in the levels of pro-inflammatory cytokines within the skin corneal layer of XLI patients compared to the control group, whether the corneal layer was lesional or non-lesional [5].

On the other hand, MPS is widely used as a screening test for rare diseases [27]. Screening tests, including neonatal screening tests, tandem mass spectrometry, and the automated auditory brainstem response test, are conducted in the neonatal period to detect diseases related to poor prognosis and mortality. As MPS technology develops and the demand for early diagnosis increases, attempts are being made to utilize newborn screening tests for MPS, such as in neonatal intensive care units [28]. Targeted panel sequencing can be used according to symptoms and is used in the clinical field, and a genetic approach to a diagnosis can be tried from the beginning of the evaluation process. Newborns with congenital diseases diagnosed early in the neonatal period can be managed through continuous care and the follow-up of related diseases, so a good prognosis can be expected. 

In this case, a genetic diagnosis based on MPS was able to diagnose XLI in the early stages after birth, and it was possible to consider the continuous treatment of skin symptoms and related disease testing. The majority of XLI patients, around 85–90%, exhibit a defect characterized by a complete deletion encompassing the entire *STS* gene and its adjacent sequences. However, about 10% of cases result from point mutations or partial deletions [25]. The current best practices for CNV detection often necessitate the use of MLPA for detecting CNVs in gene size or CMA for identifying larger CNVs exceeding 50 Kb. 

In previous studies, suspected XLI was screened initially or diagnosed molecularly by fluorescence in situ hybridization (FISH) [29,30,31] or CMA [32]. FISH can provide visual confirmation of the presence or absence of the *STS* gene or its deletion in XLI. It may be advantageous when there is a known familial deletion or when rapid results are needed. However, FISH can be more targeted and may require fewer resources than MLPA. In contrast, MLPA is a PCR-based technique that can detect CNVs across multiple genes simultaneously. It can also identify deletions, duplications, and other CNVs, including those of smaller sizes. MLPA may provide higher resolution and quantitative information about CNVs. The choice between FISH and MLPA for the genetic diagnosis of XLI may depend on various factors, including the specific objectives of the analysis, available resources, and laboratory expertise. In some cases, both techniques may be used to complement each other. FISH can be valuable for initial screening and confirming large deletions, while MLPA can provide more detailed information on CNVs and is useful for broader genetic testing. Ultimately, the decision should be made in consultation with geneticists or clinical laboratory experts who can assess the specific needs of the patient and the clinical context. 

In addition to sequencing a clinically enhanced exome to enable the targeted analysis of disease-specific variants, the implementation of CNV detection algorithms using various tools within routine targeted panel sequencing diagnostic protocols can lead to immediate improvements in clinical care for individuals affected by genetically diverse diseases [33,34]. Therefore, sequencing data generated through MPS can serve as a valuable initial step for CNV screening in genetic diagnostic settings. This screening step has the potential to enhance genetic diagnoses of hereditary skin diseases.

## 5. Conclusions

In this review, we successfully conducted a genetic diagnosis, identifying a hemizygous deletion involving the *STS* and *PUDP* genes through targeted panel sequencing with CNV analysis in a patient with XLI. The application of targeted panel sequencing based on neonatal skin symptoms proved to be an effective approach in the clinical context. Our report underscores the importance of implementing CNV screening techniques, including sequencing data analysis and gene dosage assays such as MLPA, to detect substantial deletions encompassing the *STS* gene region of Xq22 in individuals suspected of having XLI.

## Figures and Tables

**Table 1:** List of 95 genes included in the customized hereditary dermatology panel.

*AAGAB*, *ABCA12*, *ALAS2*, *ALOX12B*, *ALOXE3*, *ANAPC1*, *APCDD1*, *AQP5*, *CARD14*, *CAST*, *CDH3*, *CDSN*, *CERS3*, *COL17A1*, *COL7A1*, *CSTA*, *CTSC*, *CYP4F22*, *DSC2*, *DSG1*, *DSG4*, *DSP*, *DST*, *EDA*, *EDAR*, *EDARADD*, *ENPP1*, *EXPH5*, *FECH*, *FERMT1*, *GJA1*, *GJB2*, *GJB3*, *GJB4*, *GJB6*, *HOXC13*, *HR*, *IKBKG*, *ITGA3*, *ITGA6*, *ITGB4*, *JUP*, *KDSR*, *KLHL24*, *KRT1*, *KRT10*, *KRT14*, *KRT16*, *KRT17*, *KRT5*, *KRT6A*, *KRT6B*, *KRT6C*, *KRT74*, *KRT85*, *KRT9*, *LAMA3*, *LAMB3*, *LAMC2*, *LIPH*, *LPAR6*, *LSS*, *MSX1*, *NECTIN1*, *NECTIN4*, *NFKB2*, *NFKBIA*, *NIPAL4*, *PKP1*, *PLEC*, *PNPLA1*, *PORCN*, *RHBDF2*, *RMRP*, *RSPO1*, *SDR9C7*, *SERPINB7*, *SERPINB8*, *SLC27A4*, *SLURP1*, *SNAP29*, *ST14*, *STS*, *SULT2B1*, *TAT*, *TGM1*, *TGM5*, *TP63*, *TRPV3*, *TSPEAR*, *WNT10A*, *ALDH3A2*, *C3orf52*, *SMARCAD1*, *SNRPE*

**Table 2:** Reported Korean patients with X-linked ichthyosis and variable symptoms caused by Xp22 deletion.

	Case 1	Case 2	Case 3	Case 4	Case 5	Case 6	Case 7
Reference	[4]	[6]	[6]	[7]	[7]	[7]	This study
Method	CMA	MLPA	MLPA	CMA	CMA	CMA	MPS,MLPA
Deletion size	9.7 Mb	0.720 Mb?	0.720 Mb?	1.686 Mb	1.654 Mb	1.670 Mb	0.719 Mb
Deletion range	Xpter to 9,786,297	7,108,996 to 7,828,312?	7,108,996 to 7,828,312?	6,455,151 to 8,141,076	6,473,896 to 8,127,579	6,458,939 to 8,143,509	7,108,996 to 7,828,312
Age at Dx	13.5 yr	19 yr	19 yr	7 yr	17 mo	3 yr	1 yr
Sex	Male	Male	Male	Male	Male	Female	Male
Symptoms							
Skin							
Dry skin	P	P	P	P	P	P	P
Ichthyosis	P	P	P	P	P	P	P
Eye							
Myopia	P	N	N	P	N	N	N
Strabismus	P	N	N	N	N	P	N
Macular hypoplasia	P	N	N	N	N	N	N
Hypopigmented fundus	P	N	N	N	N	N	N
Pre-dCD	N	P	P	N	N	N	N
Neurological							
Cognitive impairment	P	N	N	P	P	P	N
Delayed GMD	P	N	N	P	P	P	N
GDD	P	N	N	P	P	P	N
Epilepsy	N	N	N	P	N	N	N
IAH	N	N	N	P	N	P	N
Face							
Dysmorphism	P	N	N	N	N	N	N
Cleft lip	P	N	N	N	N	N	N
Bone & growth							
Short stature	P	N	N	N	N	N	N
Skeletal malformation	P	N	N	N	N	N	N
Scoliosis	N	N	N	P	N	N	N
Others							
CoA	N	N	N	P	N	N	N
IUGR	N	N	N	P	P	N	N
Premature birth	N	N	N	P	N	N	N

CMA, chromosomal microarray; MLPA, multiplex ligation-dependent probe amplification; MPS, massively parallel sequencing; Dx, diagnosis; Pre-dCD, pre-descemet corneal dystrophy; GMD, gross motor development; GDD, global developmental delay; IHA, infantile axial hypotonia; CoA, coarctation of aorta; IUGR, intrauterine growth retardation.

## Data Availability

Not applicable.
